# Mediators of Racial Disparities in Hospice Use During the Last Six Months of Life

**DOI:** 10.1093/geroni/igaf122.3422

**Published:** 2025-12-31

**Authors:** Zhiwei Hu, Chengming Han, Efstathia Polychronopoulou, Mukaila Raji, Yong-Fang Kuo

**Affiliations:** The University of Texas Medical Branch, Galveston, Texas, United States; UTMB Health, The University of Texas Medical Branch, Galveston, Texas, United States; UTMB Health, The University of Texas Medical Branch, Galveston, Texas, United States; University of Texas Medical Branch at Galveston, Galveston, Texas, United States; University of Texas Medical Branch, Galveston, Galveston, Texas, United States

## Abstract

Racial disparities remain a concern in hospice care use, especially during the last six months of life. Social determinants of health factors such as low income and suboptimal housing (e.g., inconvenient housing conditions: lacking elevators in multi-level building, non-private bathrooms, or living in multi-level building) impact utilization, but their roles are not fully understood. We analyzed 2015–2020 Medicare Current Beneficiaries Survey data from 1,736 deceased beneficiaries (1,418 white, 318 non-white). Mediation analyses were conducted to test whether income or housing inconvenience explained racial differences in hospice use in the last six months of life. Causal mediation analyses adjusted for confounders, and diagnostic checks ruled out multicollinearity. Overall, 48% of patients used hospice. After adjustment for age, sex, and region, non-white patients were 25% less likely to use hospice. Income partially mediated this disparity (NIE = 1.0687, p = 0.0576), accounting for approximately 24% of the total effect. Housing inconvenience exhibited a negligible mediation effect (NIE = 1.0018, p = 0.8794). In fully adjusted models, older age (OR = 1.039, 95%CI [1.031, 1.051]) and higher income (OR highest income = 1.423, 95%CI [1.091, 1.856]) were significantly associated with hospice use. Higher housing inconvenience scores were associated with 48.1% lower likelihood of hospice use (OR = 0.519, 95%CI [0.352, 0.764]). Racial disparities in hospice utilization persist during the last six months of life. Income partially mediates these differences, whereas housing inconvenience may directly influence hospice use.

